# Effects of Polycyclic Aromatic Hydrocarbons on Lung Function in Children with Asthma: A Mediation Analysis

**DOI:** 10.3390/ijerph19031826

**Published:** 2022-02-05

**Authors:** Giovanna Cilluffo, Giuliana Ferrante, Nicola Murgia, Rosanna Mancini, Simona Pichini, Giuseppe Cuffari, Vittoria Giudice, Nicolò Tirone, Velia Malizia, Laura Montalbano, Salvatore Fasola, Roberta Pacifici, Giovanni Viegi, Stefania La Grutta

**Affiliations:** 1 National Research Council of Italy, Institute for Biomedical Research and Innovation (IRIB), Via Ugo La Malfa 153, 90146 Palermo, Italy; velia.malizia@irib.cnr.it (V.M.); laura.montalbano@irib.cnr.it (L.M.); salvatore.fasola@irib.cnr.it (S.F.); viegig@ifc.cnr.it (G.V.); stefania.lagrutta@irib.cnr.it (S.L.G.); 2 Department of Earth and Marine Sciences, University of Palermo, 90123 Palermo, Italy; 3 Department of Surgical Sciences, Dentistry, Gynecology and Pediatrics, Pediatric Division, University of Verona, 37134 Verona, Italy; giuliana.ferrante@univr.it; 4 Section of Occupational Medicine, Respiratory Diseases and Toxicology, University of Perugia, 06123 Perugia, Italy; nicola.murgia@unipg.it; 5 National Center on Addiction and Doping, Istituto Superiore di Sanità, Viale Regina Elena 299, 00161 Rome, Italy; rosanna.mancini@iss.it (R.M.); simona.pichini@iss.it (S.P.); roberta.pacifici@iss.it (R.P.); 6 Regional Agency for Environmental Protection of Sicily, 90146 Palermo, Italy; gcuffari@arpa.sicilia.it (G.C.); vgiudice@arpa.sicilia.it (V.G.); ntirone@arpa.sicilia.it (N.T.); 7 National Research Council of Italy, Institute of Translational Pharmacology (IFT), Via Ugo La Malfa 153, 90146 Palermo, Italy; 8 National Research Council of Italy, Institute of Clinical Physiology (IFC), Via Trieste 41, 56126 Palermo, Italy

**Keywords:** polycyclic aromatic hydrocarbons, lung function, children, asthma, mediation analysis

## Abstract

Studies investigating the association between urinary Polycyclic Aromatic Hydrocarbons (PAHs) and asthma in children provided inhomogeneous results. We aimed to use Mediation Analysis to discover whether a link between urinary PAHs and lung function exists and if it might be ascribed to a direct or a symptom-mediated (indirect) effect in children with asthma. This single-center prospective study was conducted in Palermo, Italy, between March and July 2017 and involved 50 children with persistent mild-moderate asthma, aged 6–11 years. At each time visit (day 0, 30, 60, and 90), physical examination, spirometry, and urine collection for detection of urinary cotinine and PAHs were performed. A symptom score was computed. The sum of individually calculated molar mass of nine PAH metabolites (ΣPAH), naphthalene metabolites (ΣPAHn) and phenanthrene metabolites (ΣPAHp) were calculated. Three children withdrew from the study due to technical problems (*n* = 1) and adverse events (*n* = 2). PAHs indirect effects on FEV_1_ (ΣPAH: −0.011, *p* = 0.04; ΣPAH_n_: −0.011, *p* = 0.04; ΣPAH_p_: −0.012, *p* < 0.001) and FVC (ΣPAH: −0.012, *p* = 0.02; ΣPAH_n_: −0.0126, *p* = 0.02; ΣPAH_p_: −0.013, *p* < 0.001) were statistically significant. In conclusion, PAHs exposures have significant indirect (symptom-mediated) effects on lung function, emphasizing the role of PAHs-induced respiratory morbidity in decreasing lung function in children with asthma.

## 1. Introduction

Polycyclic aromatic hydrocarbons (PAHs) are a group of hydrocarbons originated from the incomplete combustion of tobacco, wood, coal, and fossil fuels [1]. Concentrations of both outdoor and indoor PAHs are significantly higher in the gaseous fraction (2-, 3-, and 4-ring PAHs) than in the particulate fraction (5- and 6-ring PAHs) [2]. Moreover, PAHs may be adsorbed on inhaled particulate matter (PM) surface [3]. Their wide environmental distribution poses a potentially serious hazard for human exposure [4].

Adverse effects of PAHs exposure on respiratory health have been ascribed even to prenatal exposure [5]. Conversely, no associations were found between asthma, wheeze, cough, bronchitis and levels of PAH metabolites measured in urine from children 5 year-old enrolled in a longitudinal birth cohort study [6]. A significant association between PAH and wheeze was found [7]. Other studies found a negative association between lung function and PAHs exposure [8,9,10,11,12,13].

Assessing the total internal dose of PAHs exposure through urinary sampling has been proposed as an appealing method due to its non-invasiveness [14]. Interestingly, higher urinary PAHs concentrations were found in children living in smoking homes than non-smoking homes [15], confirming that Environmental Tobacco Smoke (ETS) is to be considered a determinant of PAHs exposure in children.

Studies investigating the association between urinary PAHs and asthma in children provided inhomogeneous results [6,16]. Overall, the relationships linking the internal dose of PAHs assessed by urinary sampling to symptoms and lung function in childhood asthma are still uncertain.

In this study, we used a causal inference approach, Mediation Analysis (MA), to discover whether a link between urinary PAHs and lung function exists and if it might be ascribed to a direct or a symptom-mediated (indirect) effect in children with asthma. Cotinine, as major nicotine metabolite, was measured in children’s urine to assess possible exposure to environmental tobacco smoke and to exclude exposed children [17].

## 2. Materials and Methods

### 2.1. Study Location

The study was carried out between March and July 2017 in Palermo, Italy, a city of 673,735 inhabitants according to the 2017 registry office, located in the northwest of Sicily Island, in the Palermo Gulf, on the shores of the Tyrrhenian Sea (38°06′56″ N 13°21′41″ E). It has a Mediterranean climate characterized by hot and dry summers as well as by mild temperatures for the rest of the year. Local air pollution is mainly related to traffic emissions and domestic heating [18].

### 2.2. Environmental Data

#### 2.2.1. PM Monitored Data

Throughout the study period, we obtained daily data on 24-h mean PM_10_ concentrations from all the available monitoring stations in the city area. Analyses were performed using the municipal air quality monitoring network managed by the local environmental agency (Risorse Ambiente Palermo—RAP, Italy). PM_10_ was sampled through a beta gauge analyzer (Environment S.A. MP101M, ENVEA, Verano Brianza, Italy), certified as equivalent to the reference method in accordance with EN12341 [19].

#### 2.2.2. Outdoor PAHs Data

Throughout the study period, the extraction and analysis of PAHs in air were carried out daily in accordance with EN 15549: 2008 [20]. 500 μL of 200 labeled PAHs were added to the filters ppb in isoctane (extraction process standard) and subsequently extracted through an Accelerated Solvent Extraction system (ASE ASE-200 Dionex/ThermoFisher Scientific), using a dichloromethane: hexane mixture (1:1). The extract was reduced to a small volume (about 1 mL) with the Buchi system; subsequently, hexane was added until a volume of 5 mL was obtained. One ml of the extract was brought almost to dryness with nitrogen flow, added with 100 μL of PAH labeled 200 ppb (internal standard).

2 μL of the extract were injected into a GC-MS (ThermoFisher Scientific DSQ-Single Quadrupole) in SIM (selected ion monitoring) mode. The PAH limits of quantification were 0.01 ng·m^−3^. As part of the overall measurement of environmental pollutants, particle-bound PAH concentrations refer to a sampling time of 5 days using quartz fiber filters (ng·m^−3^). To ensure impartial analysis, blank samples of clean quartz fiber filters (laboratory blanks) were analyzed and treated as regular samples. When the blank samples were analyzed, none of the target PAHs were detected.

### 2.3. Study Design and Population

This single-center prospective study was registered in the ClinicalTrials.gov system on 22 December 2015 (NCT02636920, https://clinicaltrials.gov/ct2/show/NCT02636920 accessed on 1 September 2021). Inclusion criteria were as follows: (1) age 6–11 years; (2) male or female; (3) diagnosis of persistent mild-moderate asthma according to GINA guidelines [21], while taking regular inhaled corticosteroids (see Section 3 for details). Exclusion criteria were as follows: (1) upper or lower respiratory tract infections in the last 2 weeks; (2) severe asthma exacerbations and/or emergency visits for asthma in the last 12 months; (3) use of systemic antibiotics in the last 4 weeks; (4) immunologic and metabolic systemic diseases; (5) major malformation of upper respiratory tract; (6) active smoking.

**Figure 1:**
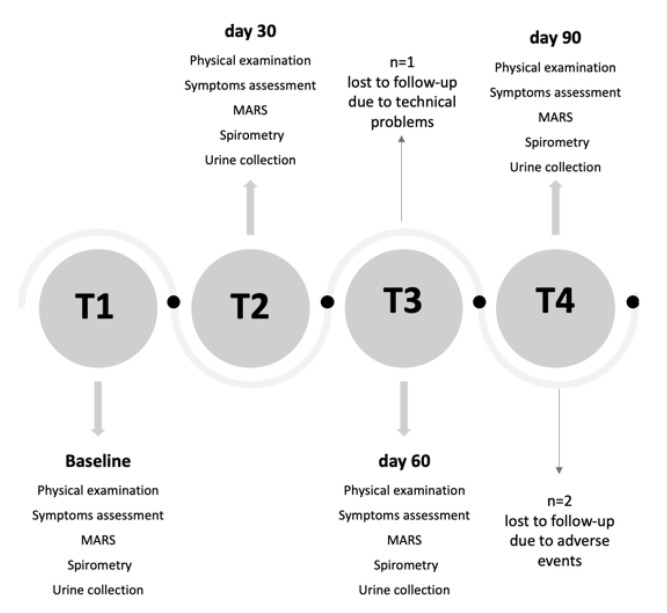
Study flow chart. MARS: Medication Adherence Rating Scale.

Sixty children were screened for eligibility. Fifty children enrolled at T1 (baseline visit) attended follow-up examinations at T2 (day 30), T3 (day 60) and T4 (day 90) (Figure 1). All children were treated with inhaled corticosteroids (ICS) at a stable dose for at least 8 weeks and during the whole study period and were provided with a mobile app (DragONE) [22], available in Italian in both iOS and Android environments, for daily recording of diurnal and nocturnal cough and wheezing, blocked nose and reliever medication use. The study was approved by the local Institutional Ethics Committee (n. 02/2017), and informed consent was obtained from all parents of each child prior to study entry. All children agreed to take part in the study, which was conducted in accordance with Good Clinical Practice and the Declaration of Helsinki.

### 2.4. Study Procedures

#### 2.4.1. Clinical Data Collection

The parents were interviewed through a modified version of the SIDRIA (*Studi Italiani sui Disturbi Respiratori nell’Infanzia e l’Ambiente*, the Italian arm of the International Study of Asthma and Allergies in Childhood—ISAAC) questionnaire, regarding parental education, maternal history of asthma and disease duration [23]. Current ETS exposure was assessed through an affirmative response to the question “Are there smokers at home?”.

Symptoms were recorded through DragONE app:CNR-ITD and CNR-IRIB, Palermo, Italy [22,24]. The symptom score was monthly computed assigning the following weights: “one” for blocked nose, “two” for diurnal cough, “three” for nocturnal cough, “four” for diurnal wheeze and “five” for nocturnal wheeze. The sum of weighed affirmative answers to questions on symptoms returned a score ranging from 0 to 15. Our symptom score assigned the highest weight to “nocturnal wheeze” because nocturnal symptoms are common events in children with asthma and their occurrence has been recognized as a clinical problem related to the severity of disease, as previously reported in children with mild to moderate asthma [25,26]. Treatment adherence was assessed through the Medication Adherence Report Scale (MARS-9) [27]. Scores for each item were summed to give a total score ranging from 9 to 45, where higher scores indicate higher levels of reported adherence to the treatment plan.

#### 2.4.2. Lung Function Assessment

Spirometry was performed at each time visit through a hand-held turbine spirometer (Pony FX portable spirometer, Cosmed, Rome, Italy) in accordance with ATS/ERS recommendations [28]. Data were expressed as a percentage of the predicted values, using the Global Lung Initiative reference values [29]. Individual spirograms are “acceptable” if they are free from artefacts, they have good starts (extrapolated volume <5% of FVC or 0.15 L) and they show satisfactory exhalation (for ≥3–6 s according to age and volume-time curve, no change in volume for ≥1 s—plateau criteria).

Out of three acceptable tests, the best forced vital capacity (FVC, i.e., the maximal volume of air exhaled with maximally forced effort from a position of full inspiration) and the maximal expiratory volume in the first second (FEV_1_, i.e., the maximal volume of air exhaled in the first second of a forced expiration from a position of full inspiration) were retained, from which FEV_1_/FVC ratio was computed as marker of airway obstruction. The forced mid-expiratory flow (FEF_25–75%_, i.e., the mean forced expiratory flow between 25% and 75% of the FVC) was selected from the manoeuvre with the largest sum of FEV_1_ and FVC. At last, FEF_25–75_/FVC ratio was also computed as marker of dysanapsis [30].

#### 2.4.3. Atopy Assessment

Atopy was defined as at least one positive (wheal ≥3 mm) skin prick tests (SPTs) to a panel of common aeroallergens (Dermatophagoides mix, cat, dog, parietaria, olive, cupressus, mixed grasses, alternaria). Atopic Index was computed as the number of positive SPTs [31] and classified as follows: 0-non-atopic, 1-one positive SPT ≥2 two or more positive SPTs.

#### 2.4.4. Urinary Cotinine Assessment

All the samples underwent a liquid chromatography-tandem mass spectrometry (LC/MS/MS) analysis of COT, revalidating for urine methodologies in use for other biological matrix [32]. In brief, calibration standards containing 1, 10, 50, 100, 500 and 1000 ng of COT and quality control (QC) samples containing 850 ng (high control), 150 ng (medium control) and 5 ng (low control) of COT were prepared in 1 mL of drug-free urine and stored at −20 °C. 500 mL of urine samples (calibration, quality control and real samples) were added with 100 ng of N-ethylnorcotinina (NENC, internal standard) and extracted twice with 1.5 mL of a mixture of chloroform-isopropanol (9:1, *v*/*v*). A 20 mL volume was injected into the liquid chromatography/tandem mass spectrometry (LC-MS/MS) system. The LC-MS/MS analyses were performed using an Alliance HPLC system (Waters, Etten-Leur, The Netherlands) interfaced to a Micromass Quattro micro API triple quadrupole mass spectrometer (Waters) equipped with an ESI probe. Chromatographic separation was achieved using a Poroshell 120, SB-C18 column (100 × 2.1 mm, 2.7 mm; Agilent Technologies, Palo Alto, CA, USA) with a mobile phase flow rate of 0.23 mL/min. The following mass spectrometry optimized conditions were used: collision energy at 18 eV for COT and 20 eV for NENC; capillary voltage at 3.0 kV, cone voltage at 25 V, source temperature at 120 °C, and desolvation temperature at 400 °C. The cone and desolvation gas flows were set at 50 and 400 L/h, respectively. The collision gas was argon at a collision cell pressure of 0.25 Pa (2.5 × 10^−3^ mbar). The transitions chosen for each compound were: *m*/*z* 177 > 80 and 177 > 98 for COT and m/z 191 > 120 and 191 > 80 for NENC. The transitions shown in bold face were used for compound quantification.

#### 2.4.5. Urinary PAHs Measurement

For PAHs analysis, the first samples of urine were collected monthly and kept frozen at −30 °C in 15 mL polypropylene tubes until the analysis. Before the analysis, urine samples underwent enzymatic hydrolysis of urinary conjugates and solid-phase extraction on C18 cartridges. Afterwards, PAHs metabolites (1-Hydroxynaphthalene, 2-Hydroxynaphthalene, 2-Hydroxyfluorene, 1,9-Hydroxyphenanthrene, 2-Hydroxyphenanthrene, 3-Hydroxyphenanthrene, 4-Hydroxyphenanthrene, 1-Hydroxypyrene) were analyzed by liquid chromatography/tandem mass spectrometry (LC-MS/MS) [33]. The LOD of PAHs ranged across the different metabolites between 0.002 ng/mL and 0.033 ng/mL. The PAHs concentration was finally corrected for urinary creatinine to reduce the influence of a diurnal pattern of PAHs metabolites often seen for urinary spot samples and related to different water consumption throughout the day [34]. In addition, three new variables were created: the sum of individually calculated molar mass of all nine PAH metabolites (ΣPAH), the sum of the naphthalene metabolites (ΣPAH_n_) and the sum of the phenanthrene metabolites (ΣPAH_p_). PAHs metabolites concentration were expressed in µg/g creatinine.

### 2.5. Statistical Analyses

Data were expressed as mean and standard deviation (SD) or as n. (%). Generalized linear mixed regression models were computed for evaluating the variable trend over time. Missing data were imputed using the Last Observation Carried Forward (LOCF), a method of imputing missing data in longitudinal studies. If a subject drops out from a study before it ends, then his or her last observed value of the dependent variable is used for all the subsequent missing observations [35].

The repeated measurements correlation was used for assessing correlations among PAHs, spirometry and symptom score over time [36]. The effects of PAHs on lung function adjusted for sex (Female vs Male), age and height were estimated via generalized mixed model as follows:

$$lung\;function_{ij}=\mu +b_{j}+\beta PAH_{ij}+\gamma symptom\;score_{ij}$$

where $lung\;function_{ij}$ is the outcome value in subject i at time visit j, and $b_{j}$ is a subject-level, normally distributed random intercept with 0 mean and variance equal to $\sigma_{b}^{2}$.

The effects of PAHs on symptom score were estimated via generalized mixed model with Poisson family. Since the symptom score is bounded from 0 to 15 and it represents a weighted number of symptoms, Poisson regression was used for modelling the mediator:

$$symptom\;score_{ij}=\eta +c_{j}+\delta PAH_{ij}$$

where $symptom\;score_{ij}$, is the outcome value in subject i at time visit j, and $c_{j}$ is a subject-level, normally distributed random intercept with 0 mean and variance equal to $\sigma_{c}^{2}$.

The extent to which the relationship between PAHs and lung function parameters could be ascribed to an independent (direct) effect and/or to a symptoms-mediated (indirect) effect was explored through Mediation Analysis (MA). MA allows assessing whether the relationship between a predictor and an outcome can be explained by their relationship with a third, mediator variable. MA was carried out using the mediation R package. Overall, our statistical framework allowed to estimate the average direct effect (ADE) and the average conditional mediated effect (ACME) (indirect effect).

Statistical power was assessed according to Pan et al. [37] A sensitivity analysis excluding children exposed to ETS, i.e., children with a urine cotinine concentration >1.1 ng/mL, was carried out. A *p*-value <0.05 was considered significant. Statistical analyses were performed using R (4.0.2, R Foundation for Statistical Computing: Vienna, Austria, 2020).

## 3. Results

### 3.1. Environmental Exposure Profile Levels

PAHs exposure and PM_10_ measured by pollutant monitoring stations of Palermo city showed a decreasing trend over the study period (Table S1 and Figure S1). A monthly decreasing trend was also observed for humidity and wind speed; conversely, an increasing trend was observed for temperature (Figure S1).

### 3.2. Characteristics of Study Population

Table 1 shows the characteristics of children at T1. Thirty-four per cent of children were females, 16% were exposed to ETS. Ninety-two per cent of children were sensitized to at least at one aeroallergen. Median ICS dose was 200 μg·day^−1^. Distribution of children visits by months is reported in Figure S2. Three children were lost to follow-up due to technical problems (*n* = 1) and withdrew from the study due to adverse events (*n* = 2). Missing data were imputed using the LOCF approach.

### 3.3. Symptoms, Urinary PAHs and Spirometry Parameters over Time

Table 2 reports symptoms, reliever use and MARS score along the four visits. Percentages of diurnal cough, diurnal and nocturnal wheeze, blocked nose and symptom score significantly decreased. Moreover, a significant decrease in the use of reliever medication was recorded.

Table 3 reports urinary PAHs and spirometry parameters along visits. Urinary 2-Hydroxynaphthalene, 2-Hydroxyfluorene, ΣPAH and ΣPAH_n_ showed a significant decreasing trend, while 1-Hydroxynaphthalene, 3-Hydroxyphenanthrene, 2-Hydroxyfluorene, 1-Hydroxypyrene and ΣPAH_p_ exhibited a borderline decreasing trend. FEV_1_ % predicted, FEF_25–75_ %predicted and FEV_1_/FVC % predicted showed a significant increasing trend and FVC %predicted a borderline increasing trend.

### 3.4. Correlations between Urinary PAHs, Spirometry Parameters and Symptom Score

Table 4 reports the repeated measure correlations of urinary PAHs, spirometry parameters and symptom score. 2-Hydroxyphenanthrene was negatively correlated with FEV_1_ and with FVC. 3-Hydroxyphenanthrene was negatively correlated with FEV_1_ and with FVC. ΣPAH_p_ was negatively correlated with FEV_1_ and with FVC. 2-Hydroxyfluorene was negatively correlated at borderline level with FEV_1_ and FVC. 1-Hydroxypyrene was negatively correlated at borderline level with FEV_1_ and FVC. 2-Hydroxynaphthalene, 1-Hydroxynaphthalene, ΣPAH and ΣPAH_n_ were positively correlated with symptom score.

### 3.5. Multivariable Analysis

Table 5 shows the estimates from the mixed generalized regression models for each unit increase in the covariate. No significant effects of PAHs on lung function were observed. Symptom score significantly affected FEV_1_ (*p* = 0.01) and FVC (*p* = 0.01). Moreover, ΣPAH, ΣPAHn and ΣPAHp were significantly associated with symptom score (*p* < 0.001).

### 3.6. Mediation Analysis

Table 6 shows the PAHs indirect and direct effects on spirometry parameters for each interquartile-range increase. The ΣPAH and the ΣPAH_n_ indirect effects on FEV_1_ (*p* = 0.04) and FVC (*p* = 0.02) were statistically significant.

**Figure 2:**
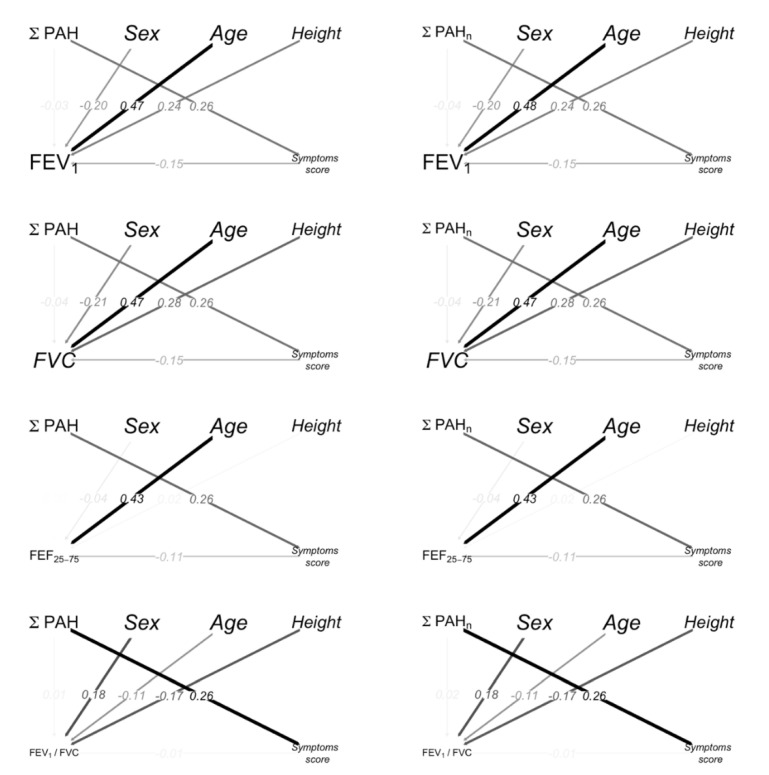
Path diagrams. The black/gray color intensity and the width of the arrows is proportional to the magnitude of the effects. The arrows from PAHs to spirometry represent the direct effects. The path from PAHs to spirometry through symptom score highlights the indirect effects. The arrows are labeled with the standardized coefficients of the Mediation Analysis. The standardized coefficients represent the degree of change in the outcome variable for every 1-unit of change (for quantitative predictors) or switching from one category to the other (for dichotomous predictors) in the predictor variable.

The path diagrams for spirometry parameters, PAHs, age, sex (Female vs Male) and height are shown in Figure 2; the color intensity and the width of the arrows are proportional to the magnitude of the effects. The path from PAHs to FEV_1_ through a symptom score indicates the indirect effects. Sex (Female vs. Male), height and age had a significant direct effect on FEV_1_.

### 3.7. Sensitivity Analysis

No difference was found between children exposed and not exposed to ETS (Table S2). Table S3 reports urinary PAHs and spirometry parameters along visits. A borderline significant decreasing trend was observed for 2-Hydroxyfluorene, ΣPAH and ΣPAH_n_. Table S4 reports symptoms along visits of children not exposed to ETS (*n* = 42). A significant decreasing percentage of diurnal cough, nocturnal wheeze, blocked nose and symptom score was observed. The path diagrams for the MA on children not exposed to ETS are reported in Figure S3.

### 3.8. Power Analysis

A sample size of 50 subjects with four repeated measurements for each subject yields about 80% power considering an observed intraclass correlation coefficient of 0.15, an effect of 0.26 of the independent variable on the mediating variable and an effect of 0.15 of the mediating variable on the dependent variable adjusted for the independent variable.

## 4. Discussion

We evaluated through a mediation analysis the relationship among urinary PAHs, symptoms and lung function in children with asthma treated with ICS therapy at a stable dose, showing that both ΣPAH and ΣPAH_n_ have significant indirect (symptom-mediated) effects on FEV_1_ and FVC, even at low air concentrations. A novel symptom score was applied, in the absence of standardized criteria for overall symptom scoring. In particular, we chose to weigh our symptom score assigning the highest weight to “nocturnal wheeze” given that nocturnal symptoms are common in children with asthma and their occurrence has been recognized as a clinical problem related to the severity of disease, as previously reported in children with mild to moderate asthma [25,26].

Throughout the study period, a significant reduction of diurnal cough, blocked nose and symptom score was observed, along with a significant decreasing use of reliever medication. This finding is not surprising, given that all the participants were on a stable continuous ICS therapy and were frequently visited. Moreover, a steady level of self-reported adherence to controller therapy was observed throughout the study period.

Interestingly, a concurrent significant decreasing trend was observed for urinary levels of 2-Hydroxynaphthalene, ΣPAH and ΣPAH_n_, while a decreasing trend was observed for 2-Hydroxyfluorene at the borderline level. A similar decreasing trend was observed for outdoor PAHs level. Overall, these results suggest that a low level of outdoor PAHs may be reflected by a decreasing PAHs internal dose measured in the urinary samples collected during the study period.

Indeed, urinary levels of 2-Hydroxynaphthalene, 1-Hydroxynaphthalene and 3-hydroxyphenanthrene was proposed as surrogates of PAHs exposure [38]. More recently, a study assessed a stove intervention program on non-smoking adults using urinary 2-naphthol as a biomarker for inhaled PAH exposure, reporting significantly lower levels in the intervention group (chimney-equipped stoves) than in controls (open-fire stoves) [39].

It should be pointed out that outdoor PAH levels measured in the current study were relatively low, probably due to the lack of industrial sites around the urban study site; therefore, the main sources of PAHs were traffic and domestic heating, which is generally rarely used given the mild temperatures that characterize our study location for most of the year. Indeed, PAHs levels measured in the city of Palermo are below the air quality standards for the protection of health, as given in the EU Ambient Air Quality Directives (BaP target value: 1 ng/m^3^) [40] and are in line with the estimated reference levels by WHO (0.12 ng/m^3^) (Evolution of WHO air quality guidelines: past, present and future. Copenhagen: WHO Regional Office for Europe; 2017).

Outdoor PAHs concentration in the city of Palermo was quite similar to the median value measured in the background region of southern Bohemia where primary sources of airborne PAHs included domestic heating and automobile exhaust emissions (1.1 ng/m^3^; interquartile range 0.6–1.7 ng/m^3^) in the study by Choi et al., whereas PAHs levels measured in the high exposure district, characterized by intensive industrial activities, was approximately 8-times higher [41]. Interestingly, prevalence rates of childhood asthma in these two monitoring sites were ~7.9% and 30%, respectively, i.e., higher than the 4.2% prevalence of current asthma detected in the years 2006/2007 through a cross-sectional study conducted in a random sample of children-adolescents, aged 10–17 years, living in the city of Palermo [42].

In a study on newborns of non-smoking mothers, the effect of in utero exposure to PAHs on respiratory health during the first 2 years of life was positively associated with levels of PAH-DNA adducts measured in umbilical cord blood, suggesting that the wheezing attributable to prenatal exposure is strongly associated with the total absorbed dose of PAH in pregnancy [5]. However, these results cannot be compared to ours given the differences in study population, timing of exposure, and biological samples used to reflect the cumulative dose of absorbed PAHs.

Studies investigating the association between urinary PAHs and asthma in children provided various results. In the current study, we found significant negative correlations among 2-Hydroxyphenanthrene, 3-Hydroxyphenanthrene, ΣPAH_p_ and FEV_1_ and among 2-Hydroxyphenanthrene, 3-Hydroxyphenanthrene and FVC, as well as borderline negative correlations among 2-Hydroxyfluorene, 1-Hydroxypyrene, FEV_1_ and FVC.

An inverse association between urinary concentrations of 2-hydroxyfluorene and spirometry parameters, like FEV_1_ and FVC, was previously observed among Mexican schoolchildren [43]. Moreover, increased urinary PAHs were significantly associated with impaired FEV_1_ and FEF_25–75%_ in children living near an industrial area, regardless of asthma status [16]. However, given the relevant different study locations and levels of environmental pollutants, our results may not be properly compared to such findings.

In addition, we found significant positive correlations between symptom score and 2-Hydroxynaphthalene, 1-Hydroxynaphthalene, ΣPAH and ΣPAH_n_. Conversely, no association between urinary PAHs and asthma or respiratory symptoms in children was reported in an inner-city birth cohort in New York City [6]. Again, differences in study design, population, and air pollution levels may account for the different findings with respect to our study.

The relationships linking the internal dose of PAHs assessed by urinary sampling to symptoms and lung function in childhood asthma are, therefore, uncertain. Herein, we used MA to discover whether a link between urinary PAHs and lung function exists and if it might be ascribed to a direct or a symptom-mediated (indirect) effect in our study population. We found significant indirect effects of ΣPAH and ΣPAH_n_ on FEV_1_ and FVC. For each unit increase in ΣPAH, the symptom score increased, while, for each unit increase in the symptom score, FEV_1_ and FVC decreased. Instead, we did not observe a significant direct effect of ambient PAHs exposure on lung function. Therefore, the finding of significant symptom-mediated effects emphasizes the relevant role of PAHs-induced respiratory morbidity in decreasing lung function in children with asthma. To confirm the robustness of our findings, we performed a sensitivity analysis by excluding children exposed to ETS.

There are several limitations in the current study. First, data describing the environmental sources of PAHs in the indoor setting were not available. Moreover, individual exposure to outdoor pollution (PM and PAHs) was not available. Although outdoor air pollution was the focus of this study, exposures to indoor air pollutants may have had a substantial impact on PAHs internal dose. Nonetheless, we provided an objective evaluation of ETS exposure, which is one of the main sources of indoor PAHs exposure, by means of urinary cotinine. Second, potential recall bias could occur in participants’ self-reporting of their symptoms. However, symptoms could be recorded within a time span no longer than 24 h on the mobile app; thus, the recall bias is unlikely. Moreover, in order to validate the novel symptom score, further studies investigating the relationship with other validated tools such as C-ACT, ACQ or CARAT are required. However, as an internal validation, we have computed a correlation for repeated measures of our symptom score with the one of C-ACT and we found a significant correlation (repeated measures correlation: −0.349, *p*-value < 0.001). Further longitudinal studies are required in order to take into account seasonality. Finally, the study had a small number of participants. Larger studies would increase the power of the analyses. However, a sample size of 50 subjects and four repeated measurements for each subject yields about 80% power considering an observed intraclass correlation coefficient of 0.15, an effect of 0.26 of the independent variable on the mediating variable, an effect of 0.15 of the mediating variable on the dependent variable, adjusted for the independent variable [37].

The main strength of the current study is the longitudinal design, enabling us to collect repeated measurements on the same individuals. In addition, to our knowledge, this is the first study that used MA to estimate the effect of PAHs exposure on lung function in children with asthma. Table S5 reports the estimates of the PAHs exposure on lung function, symptoms and diseases by study design.

## 5. Conclusions

In conclusion, we showed that PAHs exposures have significant indirect (symptom-mediated) effects on lung function even at low air concentrations, emphasizing the relevant role of PAHs-induced respiratory morbidity in decreasing lung function in children with persistent stable asthma.

## Figures and Tables

**Table 1:** Baseline characteristics of the study population.

	*n* = 50 (100%)
Female, *n* (%)	17 (34.00)
Age, years, mean (SD)	9.14 (1.58)
BMI, Kg/m^2^, mean (SD)	16.93 (3.00)
Current ETS exposure, *n* (%)	8 (16.00)
Paternal education ≥ 8 years, *n* (%)	40 (80.00)
Maternal education ≥ 8 years, *n* (%)	38 (76.00)
Maternal history of asthma, *n* (%)	11 (22.00)
Paternal history of asthma, *n* (%)	12 (24.00)
Disease duration, years, mean (SD)	5.40 (2.93)
Atopic index	
0	4 (8.00)
1	14 (28.00)
≥2	32 (64.00)
Median ICS dose (fluticasone propionate) μg·day^−1^	200 (100–500)

Data are presented as *n* (%), mean (SD) or median (IQR) and were computed based on the total number of non-missing cases; *p*-values were calculated using a *t*-test or a Chi-squared test; ETS: environmental tobacco smoke; ICS: inhaled corticosteroids.

**Table 2:** Symptoms, reliever use and Medication Adherence Rating Scale (MARS) at each time visit during the study period (March and July 2017).

	T1 (Baseline Visit)	T2 (Day 30)	T3 (Day 60)	T4 (Day 90)	*p*-Value **
*n*	50	50	50 *	50 *	
Diurnal cough, *n* (%)	14 (28.00)	10 (20.00)	7 (14.00)	4 (8.00)	**<0.001**
Nocturnal cough, *n* (%)	4 (8.00)	1 (2.00)	4 (8.00)	1 (2.00)	*0.058*
Diurnal wheeze, *n* (%)	5 (10.00)	1 (2.00)	1 (2.00)	3 (6.00)	**<0.001**
Nocturnal wheeze, *n* (%)	5 (10.00)	1 (2.00)	2 (4.00)	2 (4.00)	**0.004**
Blocked nose, *n* (%)	24 (48.00)	18 (36.00)	14 (28.00)	9 (18.00)	**<0.001**
Symptom score, mean (SD)	2.18 (3.51)	1.00 (1.93)	1.08 (1.98)	0.84 (2.71)	**<0.001**
Reliever use, *n* (%)	5 (10.00)	1 (2.00)	1 (2.00)	0 (0.00)	**<0.001**
MARS score	43.0 (5.44)	43.7 (3.31)	43.9 (1.53)	44.1 (1.16)	*0.099*

* Three children were lost to follow-up due to technical problems (*n* = 1) or lost to follow-up due to adverse events (*n* = 2). Missing data were imputed using the LOCF approach. ** *p*-values come from generalized linear mixed regression model. *p*-values in bold are statistically significant; *p*-values in italic are at borderline level.

**Table 3:** Urinary PAHs and spirometry parameters along visits, during the study period (March and July 2017).

	T1 (Baseline Visit)	T2 (Day 30)	T3 (Day 60)	T4 (Day 90)	*p*-Value **
*n*	50	50	50 *	50 *	
Urinary PAHs					
2-Hydroxynaphthalene µg/g crea	7.97 (7.93)	7.13 (6.03)	6.77 (5.73)	4.84 (5.96)	**0.012**
1-Hydroxynaphthalene µg/g crea	4.72 (9.03)	3.84 (4.00)	2.57 (3.67)	2.70 (10.1)	*0.098*
2-Hydroxyfluorene µg/g crea	0.13 (0.10)	0.14 (0.12)	0.13 (0.13)	0.09 (0.10)	**0.013**
2-Hydroxyphenanthrene µg/g crea	0.08 (0.07)	0.10 (0.13)	0.09 (0.11)	0.06 (0.07)	0.154
3-Hydroxyphenanthrene µg/g crea	0.08 (0.08)	0.09 (0.07)	0.09 (0.08)	0.06 (0.05)	*0.087*
1,9-Hydroxyphenanthrene µg/g crea	0.12 (0.09)	0.13 (0.10)	0.14 (0.15)	0.09 (0.10)	0.196
4-Hydroxyphenanthrene µg/g crea	0.02 (0.02)	0.01 (0.02)	0.02 (0.04)	0.01 (0.02)	0.991
1-Hydroxypyrene µg/g crea	0.11 (0.09)	0.11 (0.08)	0.12 (0.10)	0.08 (0.08)	*0.078*
ΣPAH µg/g crea	13.2 (12.6)	11.6 (7.52)	9.94 (6.55)	7.92 (12.7)	**0.003**
ΣPAH_n_ µg/g crea	12.7 (12.5)	11.0 (7.35)	9.34 (6.41)	7.54 (12.4)	**0.003**
ΣPAH_p_ µg/g crea	0.30 (0.23)	0.33 (0.23)	0.34 (0.30)	0.22 (0.23)	*0.097*
Spirometry parameters					
FEV_1_ %predicted	91.6 (19.8)	98.8 (19.1)	101 (21.4)	97.6 (16.4)	**0.011**
FVC %predicted	92.9 (18.2)	99.0 (20.0)	99.4 (22.0)	97.3 (16.7)	*0.050*
FEF_25–75_ %predicted	85.3 (26.9)	94.4 (23.8)	98.0 (25.9)	92.5 (20.7)	**0.037**
FEV_1_/FVC %predicted	98.0 (7.42)	99.5 (5.92)	101 (5.34)	99.8 (5.39)	0.044

* Three children were lost to follow-up due to technical problems (no Internet coverage in the area of residence; *n* = 1) and lost to follow-up due to adverse events (accident; *n* = 2). Missing data were imputed using the LOCF approach. ** *p*-values derive from generalized linear mixed regression model. *p*-values in bold are statistically significant; *p*-values in italic are at borderline level.

**Table 4:** Repeated measure correlations of urinary PAHs, spirometry parameters and symptom score.

	FEV_1_ (*L*)		FVC (*L*)		FEF_25–75_ (*L*/*s*)		FEV_1_/FVC		Symptom Score	
	Rho	*p*-Value	Rho	*p*-Value	Rho	*p*-Value	Rho	*p*-Value	Rho	*p*-Value
2-Hydroxynaphthalene µg/g crea	−0.053	0.456	−0.082	0.246	0.051	0.473	0.114	0.107	0.160	**0.024**
1-Hydroxynaphthalene µg/g crea	−0.081	0.260	−0.076	0.285	−0.072	0.309	−0.022	0.755	0.182	**0.010**
2-Hydroxyfluorene µg/g crea	−0.126	*0.077*	−0.123	*0.084*	−0.060	0.395	0.037	0.602	0.065	0.368
2-Hydroxyphenanthrene µg/g crea	−0.162	**0.022**	−0.160	**0.024**	−0.098	0.167	0.054	0.447	0.027	0.705
3-Hydroxyphenanthrene µg/g crea	−0.146	**0.039**	−0.140	**0.048**	−0.109	0.124	0.037	0.598	0.061	0.397
1,9-Hydroxyphenanthrene µg/g crea	−0.058	0.413	−0.057	0.418	−0.048	0.501	0.014	0.800	0.013	0.856
4-Hydroxyphenanthrene µg/g crea	−0.065	0.358	−0.063	0.374	−0.020	0.774	0.014	0.838	0.025	0.722
1-Hydroxypyrene µg/g crea	−0.129	*0.068*	−0.126	*0.075*	−0.096	0.176	0.017	0.806	0.009	0.897
ΣPAH µg/g crea	−0.097	0.175	−0.112	0.115	−0.023	0.746	0.057	0.419	0.233	**0.001**
ΣPAH_n_ µg/g crea	−0.092	0.194	−0.108	0.127	−0.019	0.783	0.057	0.420	0.235	**0.001**
ΣPAH_p_ µg/g crea	−0.140	**0.048**	−0.137	*0.054*	−0.095	0.183	0.025	0.720	0.037	0.606
Symptom score	−0.136	*0.094*	−0.160	**0.049**	−0.047	0.563	−0.029	0.722		

*p*-values in bold are statistically significant; *p*-values in italic are at borderline level.

**Table 5:** Estimate and (SE) from generalized mixed models.

	Outcomes				
	FEV_1,_ l	FVC_,_ l	FEF_25–75,_ l/s	FEV_1_/FVC	Symptoms
Covariate					
ΣPAH µg/g crea	−0.001 (0.001)	−0.002 (0.002)	−0.002 (0.003)	−0.0001 (0.0003)	0.045 (0.007) ***
ΣPAH_n_ µg/g crea	−0.002 (0.001)	−0.002 (0.002)	−0.002 (0.003)	−0.0001 (0.0003)	0.046 (0.007) ***
ΣPAH_p_ µg/g crea	0.0003 (0.077)	0.030 (0.079)	−0.055 (0.140)	−0.015 (0.016)	1.1907 (0.381) **
Symptoms	−0.016 (0.007) **	−0.018 (0.007) **	−0.016 (0.013)	−0.0003 (0.001)	

Models for lung function were adjusted for sex, age and height. Each model contains only one covariate at time. Significant codes: 0 ‘***’ 0.001 ‘**’ 0.01.

**Table 6:** Mediation Analysis (*n* = 50): PAHs indirect and direct effects on spirometry parameters.

	Indirect Effect	*p*-Value	Direct Effect	*p*-Value	%Mediated
ΣPAH µg/g crea					
FEV_1,_ L	**−0.011**	**0.04**	−0.0210	0.38	24.6%
FVC_,_ L	**−0.0123**	**0.02**	−0.0205	0.40	27.4%
FEF_25–75,_ L /s	−0.0106	0.32	−0.0246	0.52	13.3%
FEV_1_/FVC	−0.0002	0.82	−0.00192	0.76	1.6%
ΣPAH_n_ µg/g crea					
FEV_1,_ L	**−0.0113**	**0.04**	−0.0220	0.38	24.1%
FVC_,_ L	**−0.0126**	**0.02**	−0.0219	0.38	27.8%
FEF_25–75,_ L /s	−0.0109	0.32	−0.0249	0.58	13.5%
FEV_1_/FVC	−0.00025	0.82	−0.00173	0.78	2.2%
ΣPAH_p_ µg/g crea					
FEV_1,_ L	**−0.01180**	**<0.001**	0.00263	0.94	19.1%
FVC_,_ L	**−0.01331**	**<0.001**	0.01565	0.68	6.2%
FEF_25–75,_ L /s	−0.0112	0.28	−0.0189	0.76	8.4%
FEV_1_/FVC	−0.0002	0.80	−0.0060	0.38	1.6%

*p*-values in bold are statistically significant.

## Data Availability

Data used during the current study are available from the corresponding author on reasonable request.

## Supplementary Materials

The following supporting information can be downloaded at: https://www.mdpi.com/article/10.3390/ijerph19031826/s1, Figure S1: PM10, outdoor PAHs, temperature, relative humidity and wind speed along the study period (March and July 2017) in Palermo, Italy (38°06′56″ N 13°21′41″ E); Figure S2: Distribution of children visits by months during the study period (March and July 2017); Figure S3: Path diagrams on children not exposed to ETS; Table S1: Outdoor PAHs and PM10 monitoring levels during the study period (March and July 2017) by each time-visit in Palermo, Italy (38°06′56″ N 13°21′41″ E); Table S2: Urine PAHs and spirometry parameters among No ETS and ETS children; Table S3: Urine PAHs and spirometry parameters along visits on children not exposed to ETS (*n* = 42) during the study period (March and July 2017); Table S4: Symptoms and reliever use along visits of children not exposed to ETS (*n* = 42) during the study period (March and July 2017); Table S5: Summary of estimates of the PAHs exposure on lung function, symptoms and diseases by study design.
