# Supplementary material for: Effects of Polycyclic Aromatic Hydrocarbons on Lung Function in Children with Asthma: A Mediation Analysis
Source: Int J Environ Res Public Health. 2022 Feb 5;19(3):1826. doi: 10.3390/ijerph19031826 (PMC8834823; doi:10.3390/ijerph19031826)
Supplement: Supplementary file 1 [file ijerph-19-01826-s001.zip › ijerph-1511496-supplementary.pdf]

Table S1. Outdoor PAHs and PM_{10} monitoring levels during the study period (March and July 2017) by each time-visit in Palermo, Italy (38°06′56″N 13°21′41″E).

	**T1 (Baseline Visit) N=50**	**T2 (day 30) N=50**	**T3 (day 60) N=50***	**T4 (day 90) N=50***	**p for Trend**
Benzo(a)anthracene (ng/m^3)	0.15 (0.05)	0.14 (0.04)	0.11 (0.05)	0.11 (0.04)	**0.00017**
Benzo(b)fluoranthene (ng/m^3)	0.56 (0.24)	0.35 (0.13)	0.27 (0.05)	0.29 (0.06)	**<0.001**
Benzo(k)fluoranthene (ng/m^3)	0.25 (0.09)	0.17 (0.05)	0.13 (0.03)	0.14 (0.03)	**<0.001**
Benzo(j)fluoranthene (ng/m^3)	0.19 (0.05)	0.16 (0.04)	0.12 (0.03)	0.14 (0.03)	**0.000003**
Benzo(a)pyrene (ng/m^3)	0.24 (0.11)	0.16 (0.07)	0.12 (0.03)	0.16 (0.05)	**0.0007**
Indenopyrene (ng/m^3)	0.45 (0.21)	0.25 (0.13)	0.19 (0.04)	0.23 (0.08)	**<0.001**
DiBenzo(a,h)anthracene (ng/m^3)	0.04 (0.02)	0.02 (0.01)	0.02 (0.01)	0.02 (0.01)	**0.00171**
PM_{10}	30.6 (1.19)	28.0 (2.39)	26.1 (2.72)	24.4 (0.99)	**<0.001**

*Three children were lost to follow-up due to technical problems (no internet coverage in the area of residence) (n=1) and lost to follow-up due to adverse events (accident) (n=2).

Missing data were imputed using the LOCF approach.

Figure S1. PM_{10}, outdoor PAHs, temperature, relative humidity and wind speed along the study period (March and July 2017) in Palermo, Italy (38°06′56″N 13°21′41″E). PM_{10} and outdoor PAHs were log-transformed for reducing data variability.

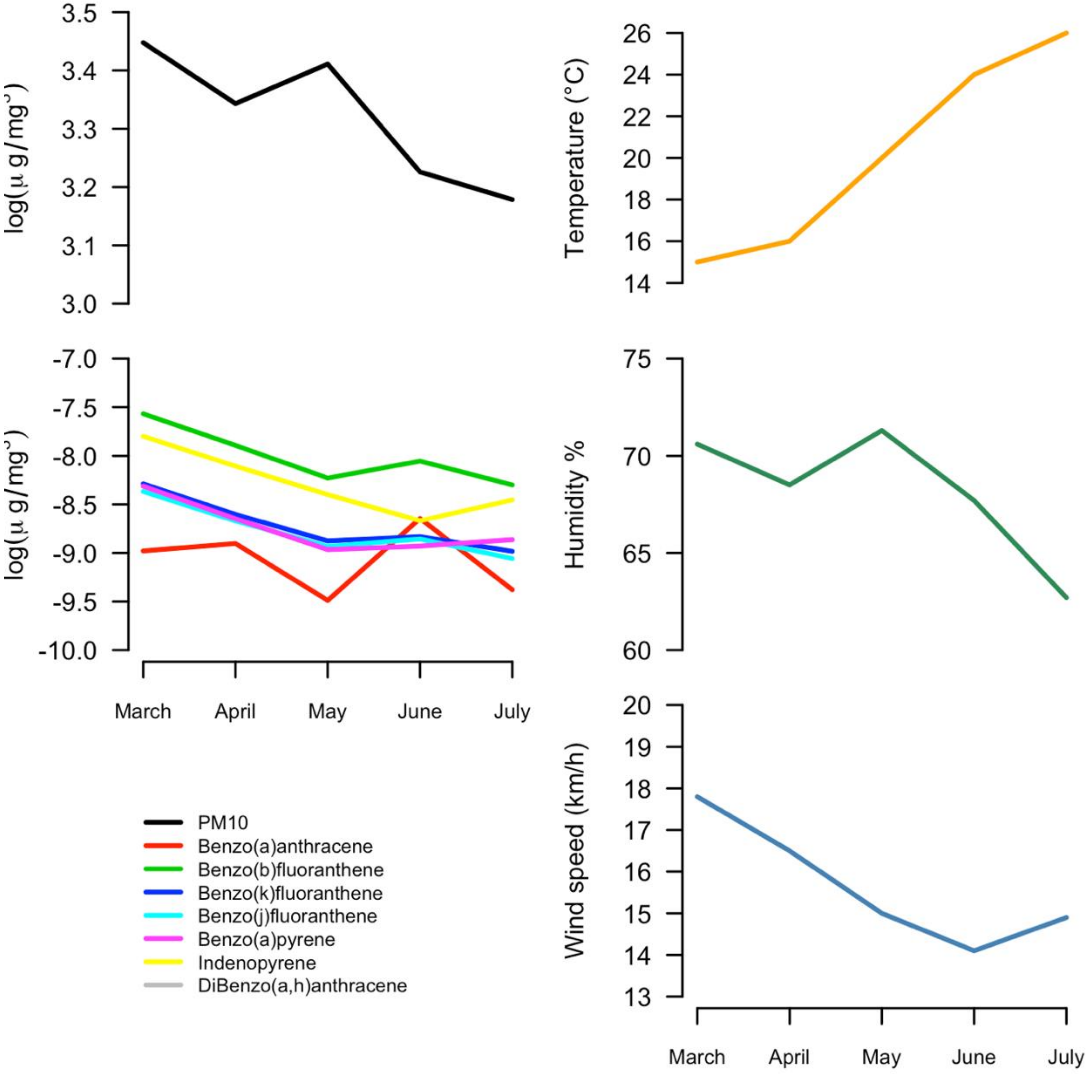

Figure S2. Distribution of children visits by months during the study period (March and July 2017).

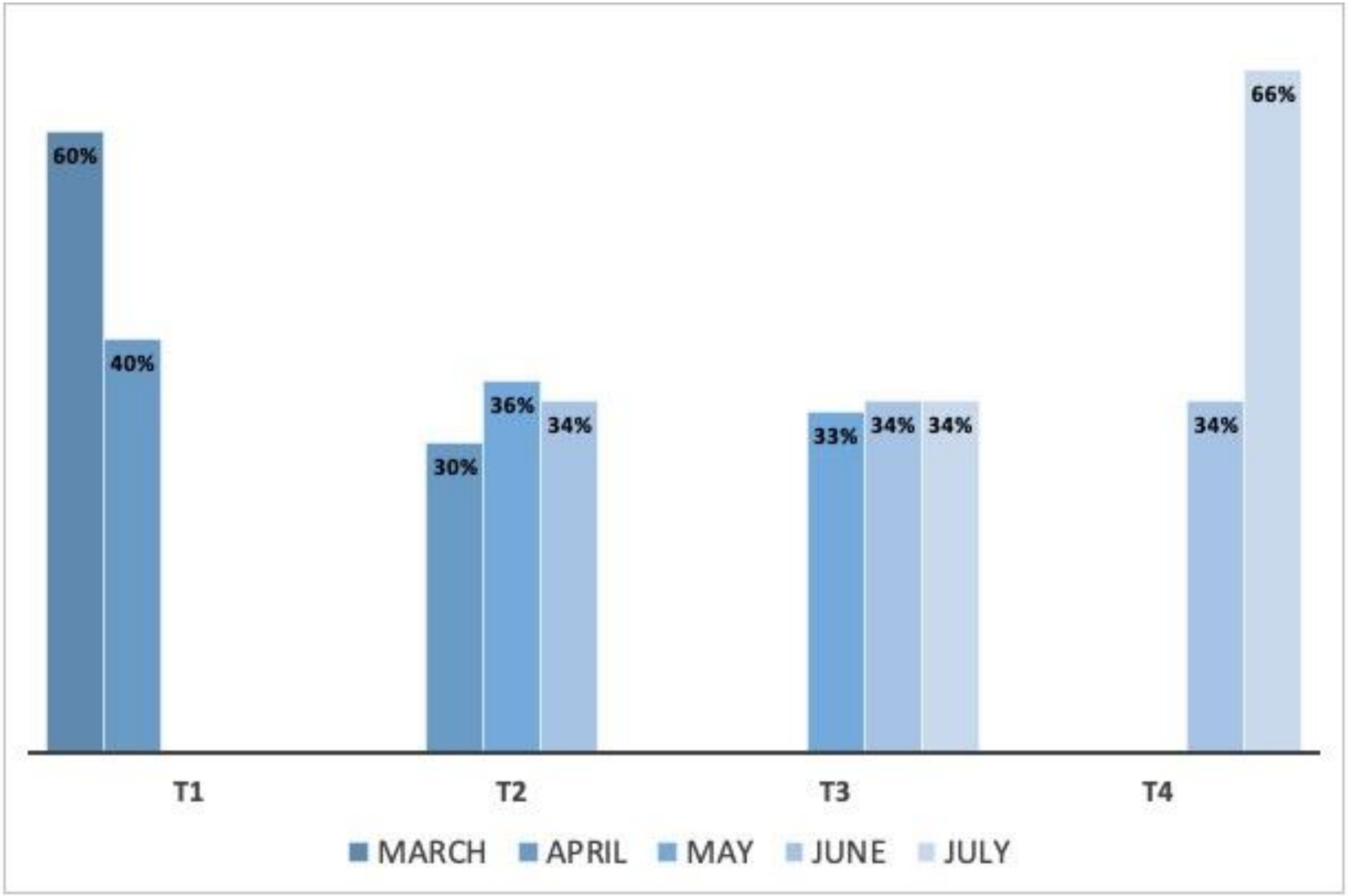

Table S2. Urine PAHs and spirometry parameters among No ETS and ETS children.

	No ETS<br>N=42	ETS<br>N=8	p-Value
Urine PAHs			
2-Hydroxynaphthalene μg/g crea	7.28 (7.02)	11.6 (11.6)	0.336
1-Hydroxynaphthalene μg/g crea	4.74 (9.72)	4.61 (4.20)	0.950
2-Hydroxyfluorene μg/g crea	0.14 (0.10)	0.09 (0.07)	0.185
2-Hydroxyphenanthrene μg/g crea	0.08 (0.08)	0.08 (0.05)	0.890
3-Hydroxyphenanthrene μg/g crea	0.08 (0.09)	0.07 (0.04)	0.557
1,9-Hydroxyphenanthrene μg/g crea	0.12 (0.10)	0.13 (0.07)	0.885
4-Hydroxyphenanthrene μg/g crea	0.01 (0.02)	0.03 (0.04)	0.377
1-Hydroxypyrene μg/g crea	0.11 (0.10)	0.11 (0.08)	0.939
ΣPAH μg/g crea	12.6 (12.6)	16.7 (13.1)	0.430
ΣPAH_n μg/g crea	12.0 (12.4)	16.2 (13.0)	0.421
ΣPAH_p μg/g crea	0.30 (0.25)	0.30 (0.16)	0.961
Spirometry parameters			
FEV_1 %predicted	93.6 (19.5)	81.0 (1 8.9)	0.114
FVC %predicted	94.7 (17.9)	83.0 (17.9)	0.121
FEF_{25-75} %predicted	87.8 (26.8)	72.2 (25.1)	0.140
FEV_1/FVC %predicted	98.2 (7.49)	96.9 (7.40)	0.663

ETS: Environmental tobacco smoke exposure

Table S3. Urine PAHs and spirometry parameters along visits on children not exposed to ETS (n=42) during the study period (March and July 2017).

	T1 (Baseline Visit)	T2 (day 30)	T3 (day 60)	T4 (day 90)	p for Trend
n	**42**	**42**	**42**	**42**	
Urine PAHs					
2-Hydroxynaphthalene µg/g crea	7.28 (7.02)	6.97 (5.96)	7.11 (5.99)	5.06 (6.36)	0.137
1-Hydroxynaphthalene µg/g crea	4.74 (9.72)	3.37 (3.41)	2.46 (3.54)	2.84 (10.9)	0.212
2-Hydroxyfluorene µg/g crea	0.14 (0.10)	0.15 (0.13)	0.14 (0.13)	0.09 (0.10)	***0.053***
2-Hydroxyphenanthrene µg/g crea	0.08 (0.08)	0.11 (0.13)	0.09 (0.12)	0.06 (0.08)	0.343
3-Hydroxyphenanthrene µg/g crea	0.08 (0.09)	0.09 (0.07)	0.10 (0.09)	0.06 (0.06)	0.163
1,9-Hydroxyphenanthrene µg/g crea	0.12 (0.10)	0.13 (0.08)	0.13 (0.10)	0.09 (0.11)	0.165
4-Hydroxyphenanthrene µg/g crea	0.01 (0.02)	0.01 (0.02)	0.02 (0.03)	0.01 (0.02)	0.731
1-Hydroxypyrene µg/g crea	0.11 (0.10)	0.11 (0.07)	0.12 (0.09)	0.08 (0.08)	0.161
ΣPAH µg/g crea	12.6 (12.6)	10.9 (7.10)	10.2 (6.69)	8.28 (13.6)	***0.060***
ΣPAH_n µg/g crea	12.0 (12.4)	10.3 (6.95)	9.57 (6.58)	7.89 (13.3)	***0.064***
ΣPAH_P µg/g crea	0.30 (0.25)	0.34 (0.23)	0.34 (0.28)	0.22 (0.24)	0.185
Spirometry parameters					
FEV_1 %predicted	93.6 (19.5)	101 (18.0)	103 (21.3)	98.5 (16.0)	0.221
FVC %predicted	94.7 (17.9)	101 (19.3)	101 (22.1)	97.9 (15.7)	0.449
FEF_{25-75} %predicted	87.8 (26.8)	97.1 (22.3)	101 (25.8)	94.6 (20.6)	0.154
FEV_1/FVC %predicted	98.2 (7.49)	99.9 (5.56)	101 (5.50)	100.0 (5.46)	0.117

Table S4. Symptoms and reliever use along visits of children not exposed to ETS (n=42) during the study period (March and July 2017).

	T1 (baseline visit)	T2 (day 30)	T3 (day 60)	T4 (day 90)	p.trend
	**N=42**	**N=42**	**N=42**	**N=42**	
Diurnal cough , n (%)	11 (26.19)	6 (14.29)	5 (11.90)	3 (7.14)	**0.015**
Nocturnal cough, n (%)	4 (9.52)	0 (0.00)	4 (9.52)	0 (0.00)	0.197
Diurnal wheeze, n (%)	2 (4.76)	1 (2.38)	1 (2.38)	1 (2.38)	0.545
Nocturnal wheeze, n (%)	4 (9.52)	1 (2.38)	1 (2.38)	0 (0.00)	**0.026**
Blocked nose , n (%)	18 (42.86)	11 (26.19)	9 (21.43)	5 (11.90)	**0.001**
Symptom score, mean (SD)	1.90 (3.60)	0.76 (1.94)	0.95 (2.01)	0.36 (1.25)	**0.007**
Reliever use, n (%)	3 (7.14%)	1 (2.38%)	1 (2.38%)	0 (0.00%)	0.068

Figure S3. Path diagrams on children not exposed to ETS. The arrows from PAHs to spirometry represent the direct effects. The path from PAH to spirometry through symptom score highlights the indirect effects. The arrows are labeled with the standardized coefficients of the Mediation Analysis. The standardized coefficients represent the degree of change in the outcome variable for every 1-unit of change (for quantitative predictors) or switching from one category to the other (for dichotomous predictors)in the predictor variable.

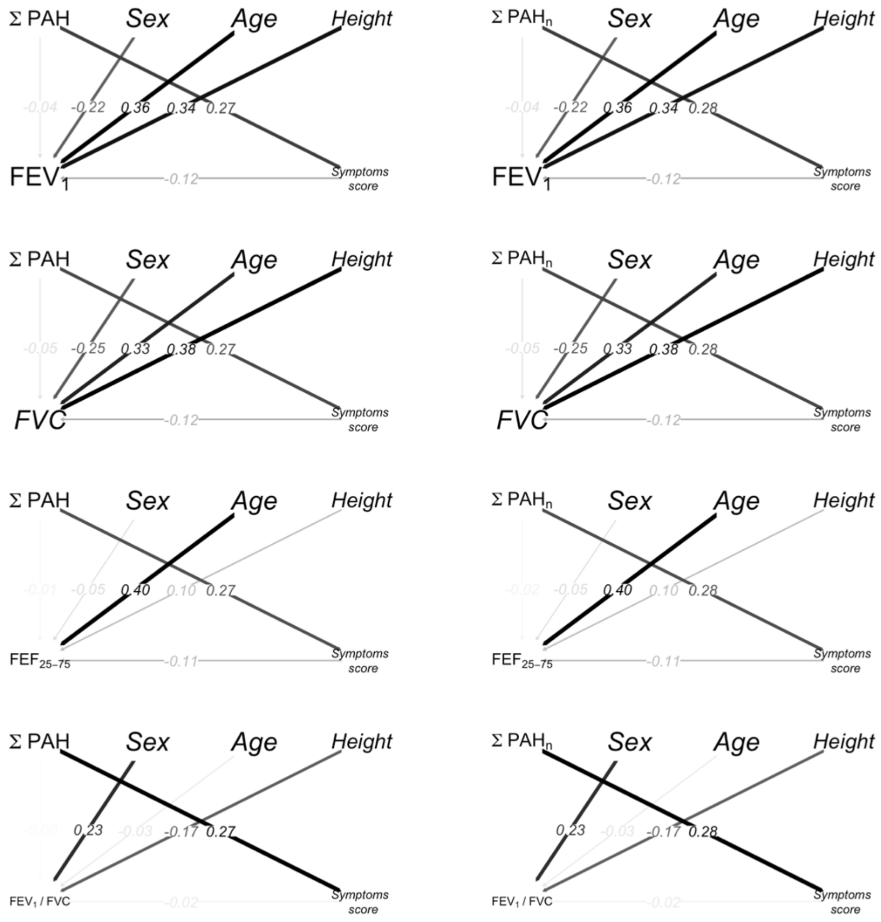

Table S5. Summary of estimates of the PAHs exposure on lung function, symptoms and diseases by study design.

Author, year	Study Population	Estimated Effect	Age (years)	Outcome	Sample Size	Country	Study Design
Miller, 2010 [6]	longitudinal birth cohort from pregnant woman affiliated with with New York Presbyterian Hospital	no association	5	asthma, wheeze, cough, bronchitis	222	USA	longitudinal
Gale, 2012 [7]	longitudinal cohort study of children with current asthma	Land Use Regression PAHs, PAH456, and phenanthrene were associated with an increased odds ranged from 1.01 (95% CI, 1.00--1.02) to 1.10 (95% CI, 1.04--1.17) of morning wheeze	6-11	wheezing	315	USA	longitudinal
Smargiassi, 2014 [8]	longitudinal study of children with asthma	Small decrease (-9.9 ml per interquartile range 95% CI: -23.4, 3.7) in respiratory function with total concentrations of PAHs measured using a backpack	7-12	FVC, FEV_1, FEF_{25-75}	72	Canada	longitudinal
Jedrychowski, 2015 [9]	longitudinal birth cohort from healthy pregnant woman	Higher residential outdoor PAH level (above 90 ng/m^3), measured using a backpack, were associated with slightly greater deficit of FEV_1 (71 mL, p=0.009)	5-9	FVC, FEV_1, FEV_{05}, FEF_{25-75}	195	Poland	longitudinal
Cakmak, 2017 [10]	General population	An interquartile change in urinary PAH metabolite was associated with significant decrements in FEV_1 (ranging from -0.56% (95% CI -0.87, -0.25) to -0.90% (95% CI -1.28, -0.53)) and FVC (up to -1.04% (95% CI -1.40, -0.68))	6 to 79	FVC, FEV_1, FEV_1/FVC	3531	Canada	longitudinal
Chen, 2018 [16]	General population	Increasing urinary PAH metabolite levels (1-OHP) were associated with decreased FEV_1(rho=−0.172, p <0.05) and $FEF_{25\%}$ (rho=−0.175, p <0.05) in children	6-12	FVC, FEV_1, FEV_1/FVC, $FEF_{25\%}$, $FEF_{50\%}$, MMF	147	China	cross-sectional
Han, 2018 [11]	General population	Among children with asthma, higher urinary PAH were significantly associated with decreased %predicted FEV_1 (by 3.5-3.8%)	6-17	FVC, FEV_1, FEV_1/FVC	2459	USA	cross-sectional
Kuang, 2020 [12]	Children with asthma and healthy childen	Each unit increase in 2-&3-OHF and 1-OHP generated -2.7 (95% CI: -4.7, -0.75, p < 0.01) and -2.3 (95% CI: -4.1, -0.58, p < 0.01) decrease in FVC	8.5 ± 2.2	FVC, FEV_1, FEV_1/FVC%, PEF, MEF, MEF_{25-75}	262	China	cross-sectional

Choi, 2021 [13]	Children with asthma and healthy childen	ambient concentration of B[a]P was associated with 4.7-times greater odds (95% CI, 1.9–11.5, p=0.001) of asthma among the non-atopic boys and with 44.8- times greater odds (95% CI, 4.7–428.2, P=0.001) among the non-atopic girls	children and adolescent	asthma	385	USA	cross-sectional
Cilluffo, 2021	longitudinal study of children with asthma	Significant indirect effects of ΣPAH and $ΣPAH_n$ on FEV_1 (p=0.04) and FVC (p=0.02)	6-11	FVC, FEV_1, FEF_{25-75}, FEV_1/FVC	50	Italy	longitudinal

Forced vital capacity (FVC); Forced expiratory in the first second (FEV_1); Forced expiratory volume in one second/forced vital capacity; FEV_1/FVC ratio; Forced expiratory volume in 0.5 seconds ($FEV_{0.5}$); Forced expiratory middle flow rate ($FEF_{25\%–75\%}$); Peak expiratory flow (PEF); Maximal midexpiratory flow (MMF); Forced mid expiratory flow ($MEF_{25\ 75}$).
